# Quantitative assessment of renal function and perfusion changes in membranous nephropathy using multiparametric magnetic resonance imaging

**DOI:** 10.1186/s13244-026-02207-6

**Published:** 2026-02-09

**Authors:** Rongchao Shi, Hao Wang, Hui Xu, Min Li, Dawei Yang, Yuxin Liu, Liting Shen, Huai Yang, Weikang Guo, Zhenghan Yang

**Affiliations:** 1 Department of Radiology, Capital Medical University Affiliated Beijing Friendship Hospital, Beijing, China; 2 Clinical Epidemiology and EBM Unit, Beijing Friendship Hospital Affiliated Capital Medical University, Beijing, China; 3 Department of Nephrology, Capital Medical University Affiliated Beijing Friendship Hospital, Beijing, China

**Keywords:** Membranous nephropathy, Multiparameter magnetic resonance imaging, Renal function, Renal blood flow

## Abstract

**Objectives:**

Renal biopsy has certain limitations for diagnosing membranous nephropathy (MN). The aim is to explore the value of MRI for diagnosing MN.

**Materials and methods:**

MN patients were divided into two subgroups based on estimated glomerular filtration rate, including the mild group and moderate to severe group. Quantitative T1 mapping and renal blood flow (RBF) of bilateral kidneys were measured, including renal cortical T1 mapping (cT1) value, medullary T1 mapping (mT1) value, cortical RBF value (cRBF), and medullary RBF (mRBF) value. The Student’s *t*-test, Mann–Whitney U test, chi-square test, and one-way analysis of variance were used.

**Results:**

Forty-seven MN patients and 54 matched healthy controls (HC) were prospectively enrolled. The cT1 and mT1 average values of HC were significantly lower than those of both MN subgroups (all *p* < 0.001) after adjusting for age and sex. Compared with the mild group and HC group, the moderate to severe group had lower cRBF (all *p* < 0.050) and mRBF average values (*p* = 0.012 and *p* < 0.001, respectively). The combination model of the T1 mapping and RBF values for differentiating MN from HC had a higher area under the curve of 0.87 (95% confidence intervals, 0.80–0.95) than single-parameter models (all *p* < 0.050), except the mT1 value model.

**Conclusions:**

Multiparametric MRI shows potential as a noninvasive adjunct tool for assessing MN, offering a possibility to guide clinical decision-making.

**Critical relevance statement:**

Multiparametric MRI provides a noninvasive approach to renal structural and perfusion changes in membranous nephropathy and offers an alternative to guide clinical treatment strategies.

**Key Points:**

- Renal biopsy has certain limitations for diagnosing membranous nephropathy, and there is an urgent need to develop a noninvasive method.
- Membranous nephropathy patients had higher cortex, medullary T1 mapping values and lower cortex, medullary renal blood flow values than healthy controls.
- Quantitative MRI parameters show potential as a noninvasive biomarker for assessing membranous nephropathy.

**Graphical Abstract:**

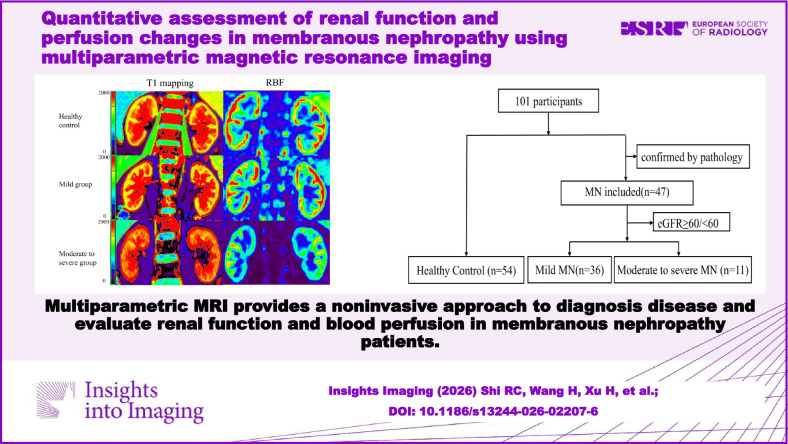

## Introduction

Membranous nephropathy (MN) is an autoimmune disease characterized by thickening of the glomerular capillary walls, resulting from immune complex deposition. With the progress of the disease, it may occur glomerular sclerosis, renal interstitial fibrosis, and decreased renal blood flow (RBF) perfusion. Recent studies indicated that the incidence of MN has been rising [1, 2], and it has become the predominant pathological type of nephrotic syndrome in adults [3]. MN has an insidious onset and clinical symptoms, posing a great challenge for early diagnosis. Moreover, the prognosis of MN patients is variable, with a third of patients experiencing spontaneous remission [4], while about 30–40% of patients exhibit poor treatment outcomes and prognosis, who would progress to end-stage renal disease within 5–15 years [5].

It is crucial for the diagnosis and treatment of disease to accurately identify the renal pathological type. Currently, renal biopsy is the gold standard for diagnosing MN, which is an invasive process, limiting its clinical applicability [6]. It is an enormous challenge for clinicians to get precise diagnosis outcomes while avoiding damage from invasive procedures. Therefore, there is an urgent need to develop an early, noninvasive, and precise diagnostic method to evaluate renal structure and function change in MN patients.

With the rapid development of magnetic resonance imaging (MRI) technology, functional MRI has shown great potential in quantifying renal functional changes [7, 8]. T1 mapping, a noninvasive and quantitative MRI technique, is used to evaluate tissue characteristics and fibrosis [9]. Previous studies have demonstrated the feasibility of T1 mapping to assess renal function and tissue fibrosis [10, 11]. And T1 is lengthened in patients compared with controls [10]. Graham-Brown et al [12], by comparing T1 mapping values between IgAN patients and healthy individuals of TI mapping, showed that T1 mapping can serve as a noninvasive method for assessing interstitial fibrosis and inflammation in IgA nephropathy and other chronic kidney diseases. And they emphasized the potential role of T1 values in IgA nephropathy and other progressive kidney diseases. Additionally, arterial spin labeling (ASL) enables noninvasive monitoring of RBF. ASL uses magnetically labeled arterial blood as an endogenous tracer. Perfusion results were measured through the subtraction of a labeled image from a non-labeled image. ASL signal demonstrated a direct linear relationship with RBF [13]. In the past few years, ASL has been widely applied in chronic kidney disease (CKD) patients, diabetic nephropathy, allografts, arterial stenosis, and renal function [14]. Mao et al showed ASL was useful for detecting underlying pathologic injury in early CKD patients with normal estimated glomerular filtration rate (eGFR) [15]. Mora-Gutiérrez et al also showed that ASL could quantify early renal perfusion impairment in diabetes mellitus, as well as changes according to different CKD stages of diabetic nephropathy [13].

Previous studies on CKD, IgA nephropathy, and diabetic nephropathy demonstrated that multiparametric MRI was a complementary or alternative method, which was able to reflect different pathophysiological information [12, 16–18]. Despite these advantages, few studies have evaluated the value of multiparametric MRI in MN. The value of multiparametric MRI to assess renal function, risk stratification, and pathophysiological features for MN remains unknown.

Therefore, this study aims to explore the value of multiparametric MRI in noninvasively assessing renal functional changes and risk stratification for MN patients. The specific objectives are as follows: (1) To analyze differences in multiparametric MRI value between MN patients and healthy controls (HC); (2) To compare changes of multiparametric MRI value in MN patients with different renal function levels; (3) To determine the correlation between multiparametric MRI value and laboratory examinations of MN patients; and (4) To evaluate the diagnostic efficacy of multiparametric MRI in distinguishing MN from HC.

## Materials and methods

### Participants

This study was approved by the Medical Ethics Committee of Beijing Friendship Hospital, Capital Medical University, and all participants provided informed consent by the Declaration of Helsinki (approval number: BFHHZS20250002).

We consecutively recruited 98 MN patients diagnosed by renal biopsy from the Department of Nephrology of our hospital from November 2023 to February 2025. Inclusion criteria were as follows: (1) adults with increased serum creatinine (Scr) and abnormal urine protein levels, and a diagnosis of CKD based on the KDIGO 2021 guidelines [19]. (2) all patients who had undergone renal biopsy and pathological results confirmed MN. (3) patients undergoing clinical assessments, laboratory examinations, and abdominal MRI. Exclusion criteria included: (1) no abdominal MRI examination; (2) poor imaging quality; (3) incomplete clinical, laboratory, or imaging data.

**Fig. 1:**
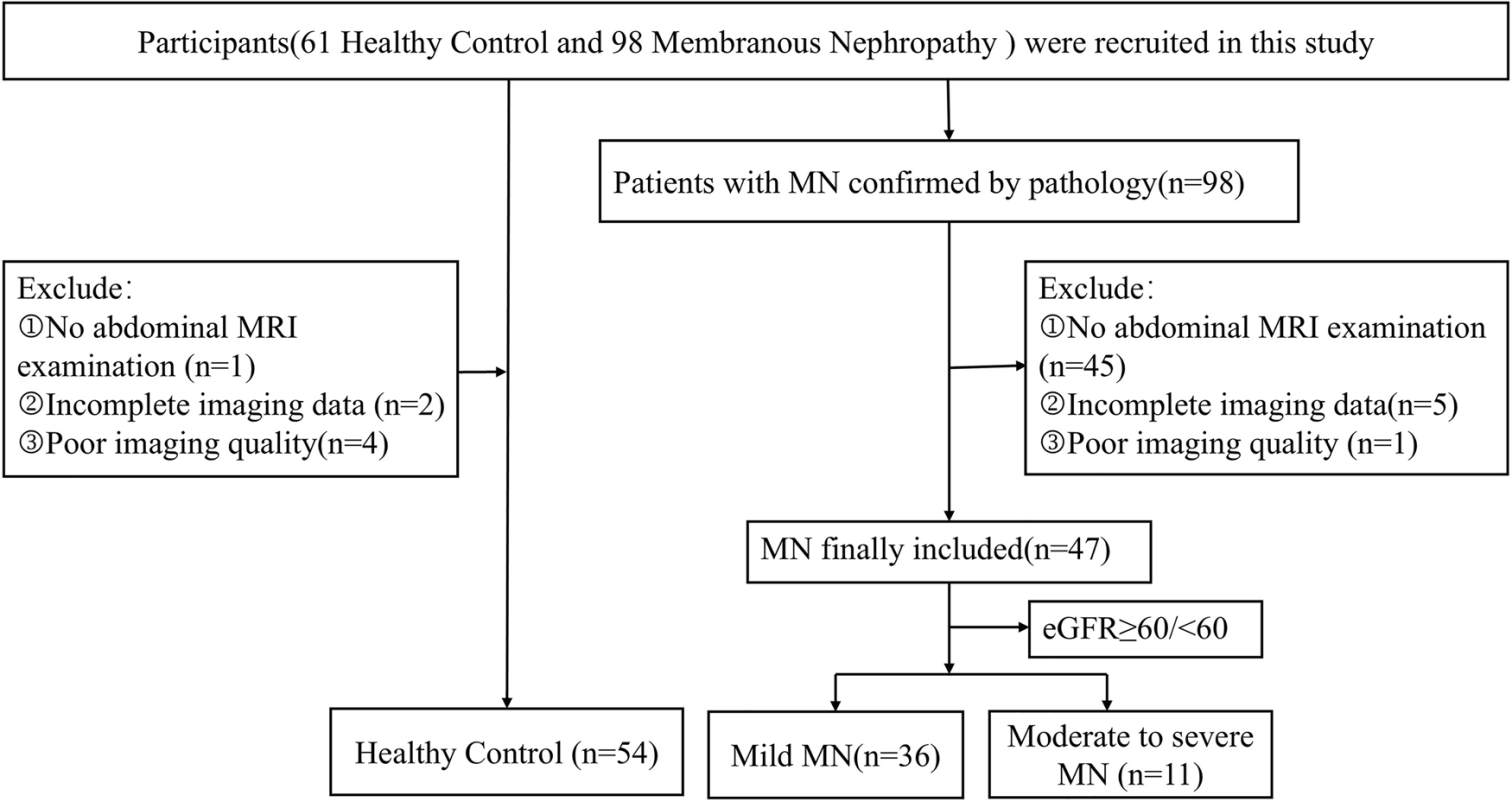
Flowchart of study participants

In addition, 61 sex-matched and age-matched healthy volunteers without kidney diseases were recruited as the HC group. Details of the study participants are presented in Fig. 1.

### Demographic data and laboratory tests

Demographic data, including sex, age, and history of hypertension and diabetes, were collected through a review of medical records during hospitalization. Laboratory examinations were obtained within 1 week before or after the MRI, including hemoglobin, Scr, eGFR, urea, uric acid, 24-h urinary protein, and anti-PLA2R antibody. The eGFR was calculated based on the CKD Epidemiology Collaboration’s equation [20]. MN patients were divided into two subgroups based on eGFR: the mild group (eGFR ≥ 60 mL/min/1.73 m^2^) and the moderate to severe group (eGFR < 60 mL/min/1.73 m^2^).

### MRI protocols

The MRI examinations were performed on a 3.0-T MRI system (Prisma, Siemens Healthineers) with an 18-channel body array coil. Patients received comprehensive safety measures and briefings before undergoing MRI scans. All participants underwent breathing training to improve image quality.

T2-weighted imaging was obtained using the half-fourier-acquired single-shot turbo spin echo and the volumetric interpolated breath-hold examination with the following parameters: orientation: coronal; repetition time (TR)/echo time (TE): 800 ms/91 ms; matrix: 320 × 320; slice-thickness: 5.0 mm; field-of-view (FOV): 360 × 360 mm.

T1-weighted imaging using the volumetric interpolated breath-hold examination had the following parameters: orientation: axial; TR/TE: 3.97 ms/2.49 ms; matrix: 260 × 320; slice-thickness: 3.0 mm; FOV: 325 × 400 mm.

T1 mapping was acquired by using the Look-Locker scheme with the following parameters: orientation: coronal; TR/TE: 3.00 ms/1.32 ms; matrix: 192 × 110; slice-thickness: 6 mm; FOV: 276 × 384 mm. The T1 maps were inline-generated.

Kidney perfusion imaging was achieved using a prototype, free-breathing, three-dimensional turbo gradient spin echo sequence with the pseudo-continuous arterial spin labeling scheme. The specific parameters were as follows: orientation: coronal; TR/TE: 5590 ms/19.28 ms; matrix: 32 × 64; slice-thickness: 5.0 mm; FOV: 150 × 300 mm. To minimize the effects of respiratory movement on perfusion imaging, retrospective registration of the image volumes was performed before averaging. The T1 of blood at 3 Tesla was 1.2 s, the blood-tissue water partition coefficient was 0.9 mL/100 g, the inversion efficiency was 0.98, and the arrival time of labeled blood was 750 ms. Inflowing arterial blood suppression and background suppression were both performed; post-labeling delay and the labeling duration were 1500 ms. Quantitative RBF maps were generated inline based on established formulas from prior studies [10, 14].

### Imaging analysis

**Fig. 2:**
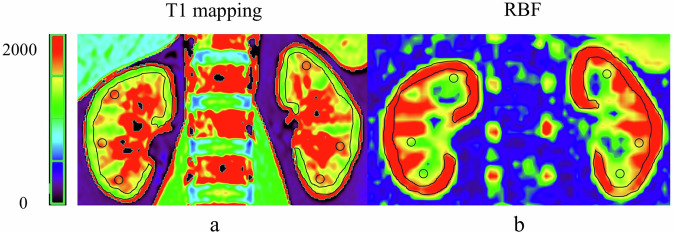
ROI selection diagram. Respective MRI images of healthy volunteers. **a** T1 maps; **b** RBF maps. RBF, renal blood flow

Two radiologists with 20 years (H.X.) and 4 years (R.C.S.) of experience in abdominal imaging diagnosis performed image analysis using a post-processing workstation (syngo via; version VE40B; Siemens Healthineers). Both readers were blinded to the clinical and pathological results of the subjects, delineated the bilateral renal cortex and medullary region of interest (ROI) at the largest level through the renal hilum. One cortical ROI (70–110 voxels) followed the outer contour of the kidney, and three medullary ROIs (12–25 voxels each) were measured in the middle of the renal parenchyma. The average value of the three renal medullary ROIs was the final medullary value. Areas with vascular structures, hemorrhage, or cysts were excluded when drawing the ROIs [15, 21]. The placement of cortical and medullary ROIs was shown in Fig. 2. The bilateral renal cortical T1 mapping (cT1) value, medullary T1 mapping (mT1) value, cortical RBF value (cRBF), and medullary RBF (mRBF) value were measured. The MRI parameter values were averaged for subsequent analyses because eGFR reflects the comprehensive function of both kidneys.

### Statistical analysis

The SPSS software version 22.0 (IBM Corp) and GraphPad Prism version 10.0 were used for statistical analysis. The intraclass correlation coefficients (ICCs) were used to evaluate the interobserver reproducibility of the MRI parameters (0.81–1.00, excellent agreement; 0.61–0.80, moderate agreement; 0.21–0.40, fair agreement; 0.00–0.20, poor agreement). The normal distribution of continuous variables was assessed by the Kolmogorov–Smirnov test. When data of two groups conformed to the normal distribution, the Student’s *t*-test was used, and data were depicted as mean ± standard deviation; otherwise, the Mann–Whitney U test was performed, shown as median and quartiles. Categorical variables were compared by the chi-square test. One-way analysis of variance was used to compare MRI parameters among the HC, mild group, and moderate to severe group. If there was a significant difference among the three groups, the multiple comparisons test was conducted to further assess differences between the two related groups. To reduce the influence of confounding factors, covariance analysis was used in all subgroup analyses. Pearson and Spearman correlation analyses examined the association between MRI parameters and clinical data. Receiver operating characteristic analyses with the area under the curve (AUC) were used to assess the diagnostic performance of multiparameter MRI for differentiating MN patients from HC. Two-tailed *p*-value < 0.05 was considered to be statistically significant.

## Results

### Clinical characteristics

In total, 54 healthy individuals (28 men, 26 women) in the HC group and 47 MN patients (28 men, 19 women) were included in this study. The MN group consists of 36 patients (22 men, 14 women) in the mild group and 11 patients (6 men, 5 women) in the moderate to severe group. There was no significant difference in sex (*p* = 0.481) among the three groups. The HC group and MN group were appropriately matched for age (52.19 ± 10.27 vs 56.30 ± 12.48, *p* = 0.086). There was a significant difference in age between the mild group and the moderate to severe group, between the HC group and the moderate to severe group (all *p* < 0.001). However, there was no difference in age between the HC group and mild group (*p* = 0.974).

We observed significant differences between the mild group and moderate to severe group in all the laboratory examination data, except for uric acid and anti-PLA2R antibody. The moderate to severe group had lower eGFR and hemoglobin than those in the mild group (all *p* < 0.001). Compared with the mild group, the moderate to severe group had higher levels of urea (*p* < 0.001), Scr (*p* < 0.001), and 24-h urinary protein (*p* = 0.018). The history of hypertension (*p* = 0.366) and diabetes (*p* = 0.952) was not statistically different in both groups. The demographic and clinical data of the study population are shown in Table 1.

**Table 1:** Demographics and clinical data

Characteristic	Mild group (*n* = 36)	Moderate to severe group (*n* = 11)	HC (*n* = 54)	*p*-value
Age^a^	52.11 ± 12.35	70.00 ± 5.76	52.19 ± 10.27	< 0.001^c^
Sex				0.481^d^
Male	22 (61.11)	6 (54.55)	28 (51.85)	
Female	14 (38.89)	5 (45.45)	26 (48.15)	
Hypertension			NA	0.366^d^
Yes	22 (61.11)	9 (81.82)		
No	14 (38.89)	2 (18.18)		
Diabetes			NA	0.952^d^
Yes	9 (25.00)	2 (18.18)		
No	27 (75.00)	9 (81.82)		
eGFR, mL/min/1.73 m^2a^	97.61 ± 14.60	43.83 ± 15.37	NA	< 0.001^e^
Hemoglobin, g/L^a^	137.28 ± 15.56	100.00 ± 16.24	NA	< 0.001^e^
Urea, mmol/L^a^	4.92 ± 1.85	8.39 ± 3.77	NA	< 0.001^e^
Serum creatinine, μmol/L^b^	69.60 (60.36–78.84)	111.70 (71.50–151.90)	NA	< 0.001^f^
24-h urinary protein, g/24 h^a^	4.27 ± 3.31	7.44 ± 5.00	NA	0.018^e^
Uric acid, μmol/L^a^	360.60 ± 97.04	369.70 ± 59.98	NA	0.771^e^
Anti-PLA2R antibody (> 20RU/mL)			NA	0.101^d^
Yes	16 (44.44)	8 (72.73)		
No	20 (55.56)	3 (27.27)		

Unless otherwise noted, data present the numbers of patients, with percentages in parentheses

*MN* membranous nephropathy, *HC* healthy control, *NA* not applicable, *eGFR* estimated glomerular filtration rate

^a^ Data are presented as means ± SDs

^b^ Data are presented as medians, with interquartile ranges in parentheses

^c^ One-way analysis of variance test

^d^ Chi-square test

^e^ Student’s *t*-test

^f^ Mann–Whitney U

### Comparison of MRI parameters

**Fig. 3:**
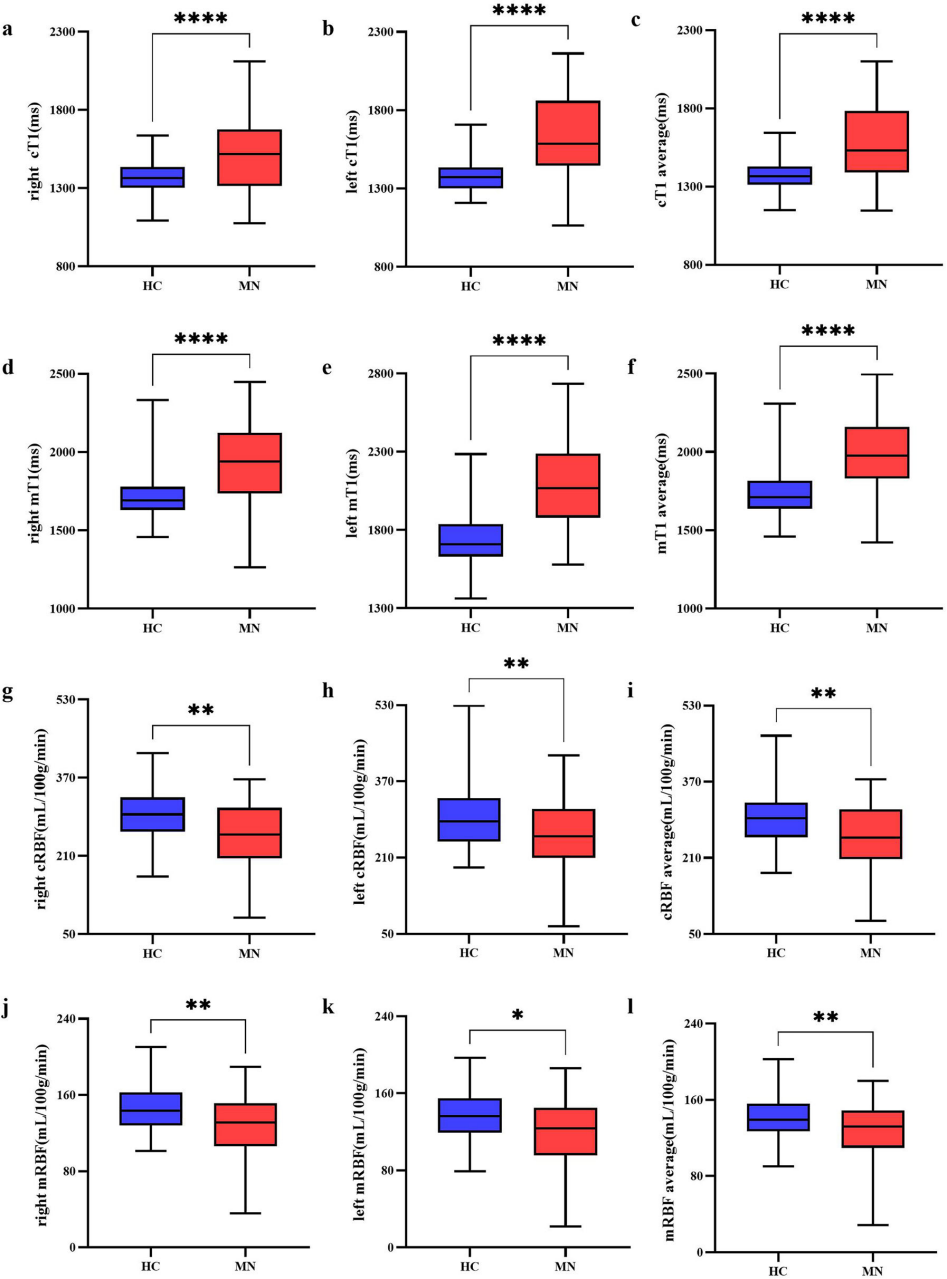
Comparison of MRI parameters between the healthy control and the MN patient group. Box plots of cT1 (**a**–**c**), mT1 (**d**–**f**), cRBF (**g**–**i**), mRBF (**j**–**l**). MN, membranous nephropathy; cT1, cortex T1 mapping; mT1, medullary T1 mapping; cRBF, cortex renal blood flow; mRBF, medullary renal blood flow. **** *p* < 0.0001, ** *p* < 0.01, * *p* < 0.05

**Fig. 4:**
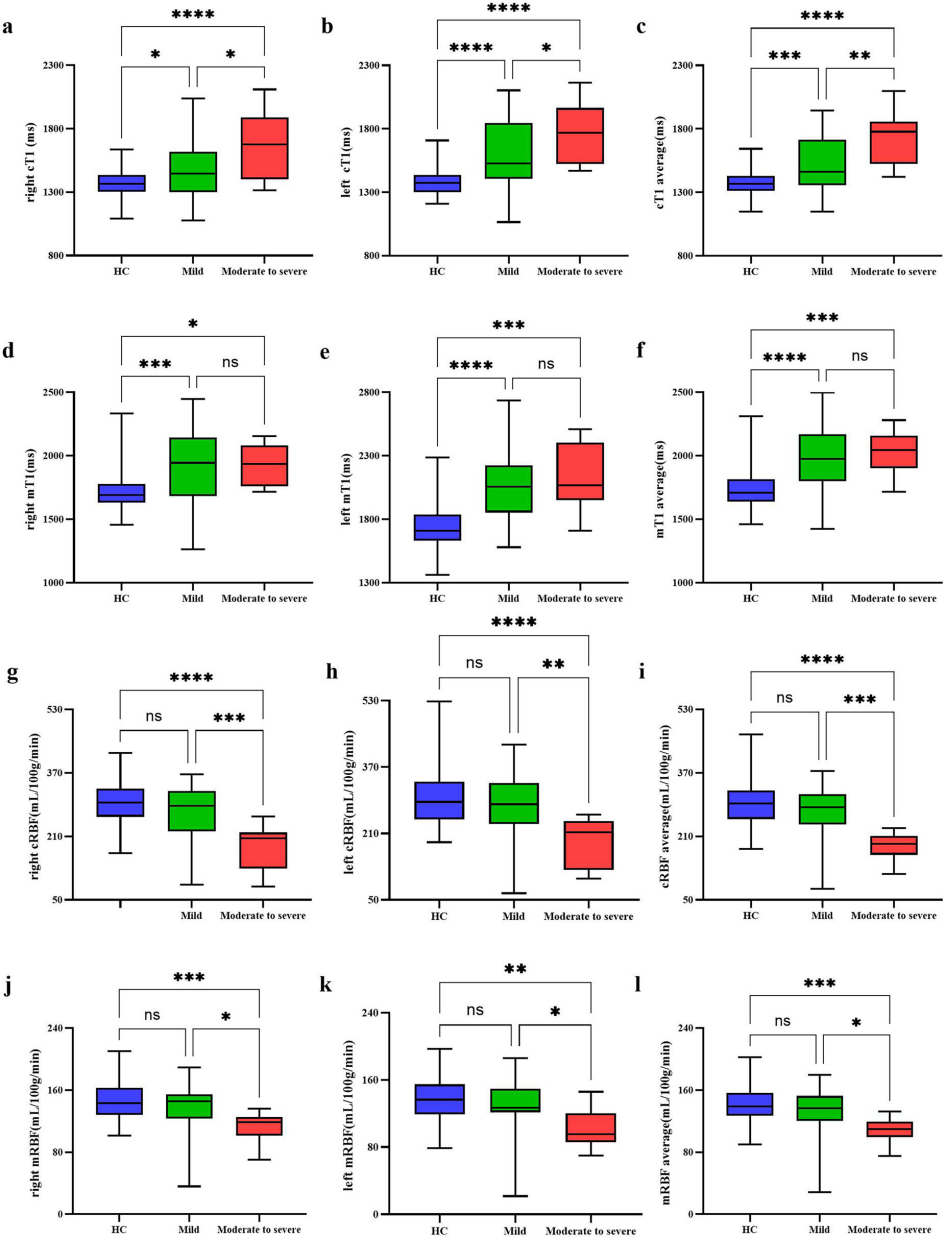
Comparison of MRI parameters among healthy control, mild, and moderate to severe groups. Box plots of cT1 (**a**–**c**), mT1 (**d**–**f**), cRBF (**g**–**i**), mRBF (**j**–**l**). MN, membranous nephropathy; cT1, cortex T1 mapping; mT1, medullary T1 mapping; cRBF, cortex renal blood flow; mRBF, medullary renal blood flow. **** *p* < 0.0001, *** *p* < 0.001, ** *p* < 0.01, * *p* < 0.05

In all subjects, the bilateral cT1 values were remarkably lower than the mT1 values (all *p* < 0.001). Compared to the HC group, MN patients had higher cT1 average value (1378.65 ± 94.64 vs 1580.94 ± 239.88, *p* < 0.001) and mT1 average value (1737.80 ± 151.91 vs 1999.96 ± 245.85, *p* < 0.001) (Fig. 3a–f). In subgroup analysis, pairwise comparisons revealed that the bilateral cT1 values had significant differences among the three groups after adjustment for age and sex (Fig. 4a–c). Compared to the HC group, the mild group and moderate to severe group had higher bilateral mT1 values (all *p* < 0.050). However, there were no significant differences in bilateral mT1 values (right *p* = 0.947; left *p* = 0.950) between the mild group and the moderate to severe group. The results are shown in Fig. 4d–f.

**Fig. 5:**
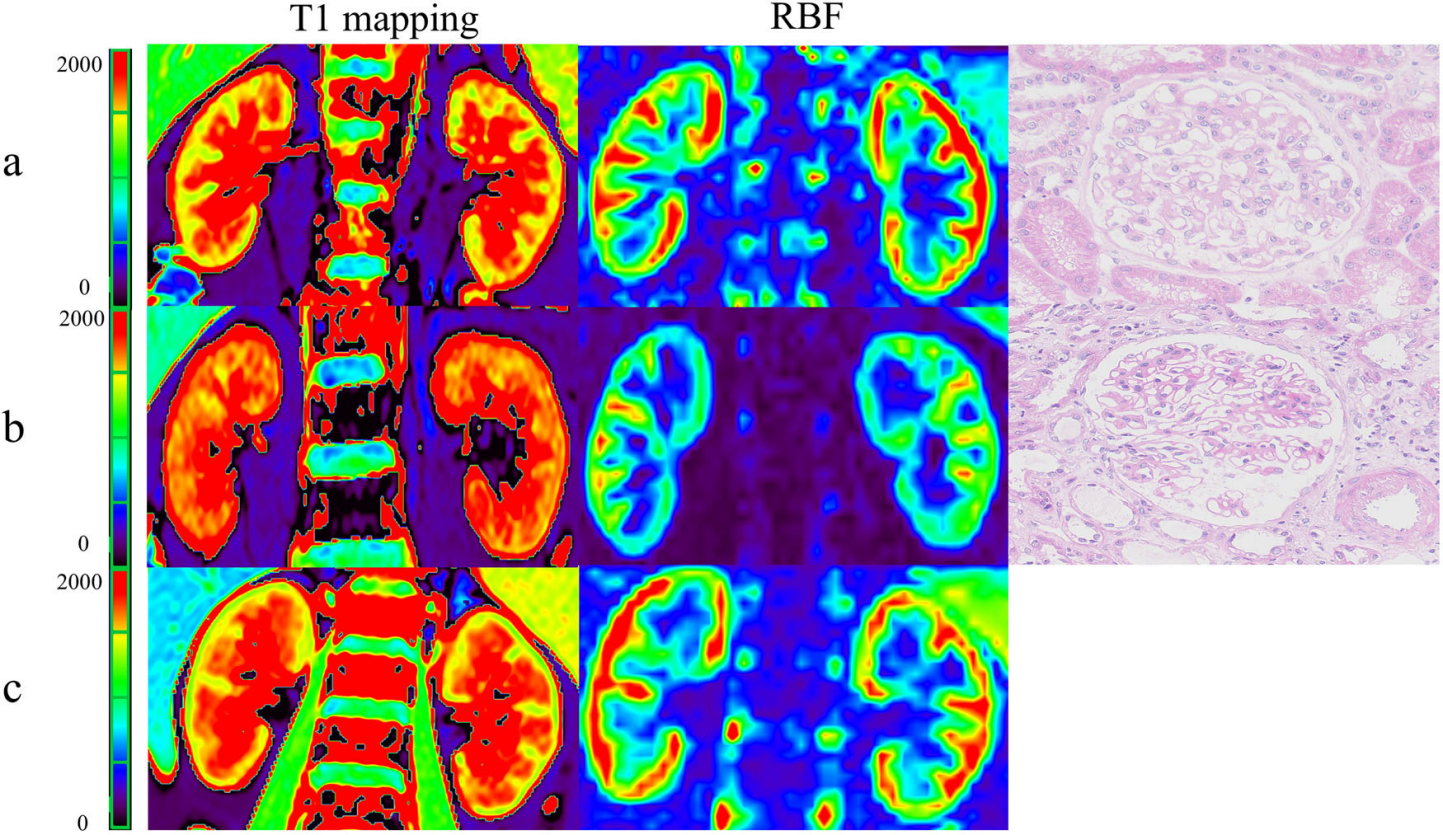
Sample graph among healthy control and MN patient groups. **a** Patients with mild group. **b** Patients with moderate to severe group. **c** Healthy control. The redder the color, the higher the T1 value and RBF value. The T1 value gradually increases as renal function decreases, and the color gradually turns red. The RBF value of moderate to severe MN is lower than that of HC and mild MN, and the red color is also less

For perfusion data, the bilateral cRBF values were significantly higher than the mRBF value in all subjects (all *p* < 0.001). MN patients had lower cRBF average value (252.95 ± 69.46 vs 295.09 ± 59.69, *p* = 0.001) and mRBF average value (126.57 ± 30.62 vs 141.62 ± 24.06, *p* = 0.007) than the HC group (Fig. 3g–l). After adjustment for age and sex, the bilateral cRBF values (all *p* < 0.010) and mRBF values (all *p* < 0.050) in the moderate to severe group were significantly lower than those of the mild group and the HC group. However, there were no significant differences in bilateral cRBF values (right *p* = 0.244; left *p* = 0.318) and mRBF values (right *p* = 0.268; left *p* = 0.364) between the mild group and the HC group (Fig. 4g–l). Typical examples of T1 and ASL parameter maps were shown in Fig. 5.

For renal anatomical length, there were no significant differences in bilateral renal lengths between the MN group and the HC group (right: 99.00 ± 11.01 vs 99.29 ± 7.59, *p* = 0.877; left: 98.04 ± 12.36 vs 99.71 ± 8.77, *p* = 0.430). In subgroup analysis, the bilateral renal lengths in the moderate to severe group were significantly shorter than those of the mild group and HC group after adjustment for age and sex (all *p* < 0.010). However, there were no significant differences in bilateral renal lengths between the mild group and the HC group (right: *p* = 0.391; left: *p* = 0.662). The detailed results were exhibited in Table 2.

**Table 2:** Multiparameter MRI values of mild MN, moderate to severe MN, and HC after adjusting for age and sex

Characteristic	Mild group (*n* = 36)	Moderate to severe group (*n* = 11)	HC (*n* = 54)	F	*p*-value^a^	*p*-value^b^	*p*-value^c^
Right renal length, mm	101.75 ± 9.74	89.97 ± 10.43	99.29 ± 7.59	7.68	< 0.001	0.391	0.005
Left renal length, mm	101.52 ± 10.99	86.63 ± 9.65	99.71 ± 8.77	10.29	< 0.001	0.662	< 0.001
Right cT1, ms	1487.35 ± 235.08	1675.02 ± 276.88	1372.05 ± 111.56	13.62	0.011	0.013	< 0.001
Left cT1, ms	1586.12 ± 273.29	1776.19 ± 238.60	1385.25 ± 111.32	23.19	0.017	< 0.001	< 0.001
cT1 average, ms	1536.74 ± 229.18	1725.60 ± 225.44	1378.65 ± 94.64	23.08	0.005	< 0.001	< 0.001
Right mT1, ms	1908.03 ± 306.42	1932.14 ± 151.89	1727.54 ± 158.94	8.84	0.947	< 0.001	0.018
Left mT1, ms	2079.94 ± 324.86	2106.84 ± 275.16	1748.06 ± 191.43	22.07	0.950	< 0.001	< 0.001
mT1 average, ms	1993.99 ± 264.21	2019.49 ± 182.09	1737.80 ± 151.91	21.23	0.929	< 0.001	< 0.001
Right cRBF, mL/100 g/min	272.69 ± 67.73	182.29 ± 56.79	293.77 ± 56.29	15.44	< 0.001	0.244	< 0.001
Left cRBF, mL/100 g/min	273.97 ± 80.92	190.23 ± 58.78	296.40 ± 67.33	10.06	0.003	0.318	< 0.001
cRBF average, mL/100 g/min	273.33 ± 64.66	186.26 ± 34.27	295.09 ± 59.69	15.32	< 0.001	0.211	< 0.001
Right mRBF, mL/100 g/min	136.64 ± 31.55	110.32 ± 20.37	145.80 ± 25.35	7.88	0.017	0.268	< 0.001
Left mRBF, mL/100 g/min	128.96 ± 33.46	102.10 ± 22.93	137.44 ± 26.50	6.94	0.022	0.364	0.001
mRBF average, mL/100 g/min	132.80 ± 31.08	106.21 ± 18.37	141.62 ± 24.06	8.41	0.012	0.269	< 0.001

*MRI* magnetic resonance imaging, *MN* membranous nephropathy, *HC* healthy control, *cT1* cortex T1 mapping, *mT1* medullary T1 mapping, *cRBF* cortex renal blood flow, *mRBF* medullary renal blood flow

^a^ Post hoc paired comparisons between the mild group and the moderate to severe group

^b^ Post hoc paired comparisons between the mild group and the HC group

^c^ Post hoc paired comparisons between the moderate to severe group and the HC group

### Correlations between MRI parameters and laboratory data

The cT1 average value had a significantly negative correlation with eGFR and hemoglobin (r: −0.43 and −0.37; *p* = 0.003 and 0.011, respectively), but there was a positive correlation with Scr and 24-h urinary protein (r: 0.29 and 0.33; *p* = 0.044 and 0.023, respectively). The mT1 average value had no significant correlation with laboratory data (all *p* > 0.050).

For perfusion data, the cRBF average value and mRBF average value had significantly positive correlation with eGFR and hemoglobin (r: 0.30 to 0.51, all *p* < 0.050). But the cRBF average value and mRBF average value had a significantly negative correlation with urea and Scr (r: −0.44 to −0.29, all *p* < 0.05). And the cRBF average value had the strongest correlation with eGFR (r = 0.51, *p* < 0.001). The detailed results were shown in Table 3.

**Table 3:** Correlation analysis between MRI parameters and laboratory examination in MN patients

	eGFR		Hemoglobin		Urea		Scr		24-h urinary protein		Uric acid	
	r	*p*-value	r	*p*-value	r	*p*-value	r	*p*-value	r	*p*-value	r	*p*-value
Right cT1	−0.41	0.004	−0.40	0.005	0.25	0.095	0.32	0.026	0.40	0.005	−0.07	0.653
Left cT1	−0.40	0.006	−0.27	0.006	0.09	0.537	0.21	0.151	0.18	0.237	−0.07	0.661
cT1 average	−0.43	0.003	−0.37	0.011	0.17	0.252	0.29	0.044	0.33	0.023	−0.07	0.624
Right mT1	−0.11	0.481	−0.05	0.719	0.09	0.529	0.06	0.683	0.15	0.323	−0.03	0.826
Left mT1	−0.17	0.263	−−0.17	0.259	0.04	0.771	0.09	0.556	0.10	0.519	−0.31	0.036
mT1 average	−0.18	0.219	−0.14	0.360	0.10	0.510	0.09	0.546	0.17	0.251	−0.21	0.152
Right cRBF	0.32	0.026	0.38	0.008	−0.28	0.061	−0.37	0.010	−0.19	0.191	−0.02	0.884
Left cRBF	0.39	0.007	0.32	0.029	−0.38	0.008	−0.39	0.006	−0.18	0.219	−0.09	0.554
cRBF average	0.51	< 0.001	0.40	0.005	−0.41	0.005	−0.44	0.002	−0.20	0.177	−0.07	0.663
Right mRBF	0.36	0.013	0.32	0.030	−0.30	0.041	−0.36	0.013	−0.16	0.277	0.03	0.842
Left mRBF	0.31	0.032	0.27	0.077	−0.27	0.069	−0.34	0.020	−0.19	0.200	−0.06	0.685
mRBF average	0.35	0.016	0.30	0.039	−0.29	0.045	−0.37	0.011	−0.16	0.281	−0.02	0.906

*MRI* magnetic resonance imaging, *MN* membranous nephropathy, *eGFR* estimated glomerular filtration rate, *Scr* serum creatinine, *cT1* cortex T1 mapping, *mT1* medullary T1 mapping, *cRBF* cortex renal blood flow, *mRBF* medullary renal blood flow

### Diagnostic efficiency of MRI parameters for differentiating MN from HC

To differentiate MN patients from the HC group, sensitivity, specificity, and AUC were calculated. The average values of MRI parameters were used to analyze. Table 4 showed the AUC values ranging from 0.63 to 0.87, with the mT1 model exhibiting a high AUC of 0.83 (95% confidence intervals (CI), 0.74–0.91), with sensitivity (76.60%; 95% CI, 62.78–86.40%), specificity (85.19%; 95% CI, 73.40–92.30%) in single-parameter models. The combination model of T1 and RBF exhibited high AUC (0.87; 95% CI, 0.79–0.95), sensitivity (87.23%; 95% CI, 74.83–94.02%), and specificity (83.33%; 95% CI, 71.26–90.98%). The AUC values of multiparametric MRI models were significantly higher than the single RBF models (all *p* < 0.001). The AUC values of multiparameter models were higher than single-parameter models, such as the combination model of cT1, mT1, cRBF, and mRBF versus the single cT1 model (*p* = 0.021); the combination model of cT1, mT1, and cRBF versus the single cT1 model (*p* = 0.016); all combination models versus single RBF models (all *p* < 0.001).

**Table 4:** Diagnostic performances of multiparameter MRI for distinguishing MN from HC

	Sensitivity	Specificity	AUC
cT1	55.32% (41.25–68.59%)	90.74% (80.09–95.98%)	0.77 (0.67–0.87)
mT1	76.60% (62.78–86.40%)	85.19% (73.40–92.30%)	0.83 (0.74–0.91)
cRBF	53.19% (39.23–66.67%)	75.93% (63.05–85.36%)	0.65 (0.55–0.76)
mRBF	38.30% (25.79–52.57%)	85.19% (73.40–92.30%)	0.63 (0.52–0.74)
cT1 + mT1	65.96% (51.67–77.83%)	90.74% (80.09–95.98%)	0.84 (0.75–0.92)
cT1 + cRBF	63.83% (49.54–76.03%)	94.44% (84.89–98.49%)	0.80 (0.71–0.90)
cT1 + mRBF	57.45% (43.28–70.49%)	96.30% (87.46–99.34%)	0.77 (0.67–0.86)
mT1 + cRBF	80.85% (67.46–89.58%)	88.89% (77.81–94.81%)	0.86 (0.78–0.94)
mT1 + mRBF	85.11% (72.31–92.59%)	74.07% (61.07–83.88%)	0.86 (0.78–0.93)
cRBF + mRBF	53.19% (39.23–66.67%)	75.93% (63.05–85.36%)	0.68 (0.57–0.78)
cT1 + mT1 + cRBF	87.23% (74.83–94.02%)	83.33% (71.26–90.98%)	0.87 (0.80–0.95)
cT1 + mT1 + mRBF	68.09% (53.83–79.60%)	92.59% (82.45–97.08%)	0.86 (0.78–0.94)
cT1 + cRBF + mRBF	63.83% (49.54–76.03%)	94.44% (84.89–98.49%)	0.80 (0.71–0.90)
mT1 + cRBF + mRBF	80.85% (67.46–89.58%)	87.04% (75.58–93.58%)	0.86 (0.79–0.94)
cT1 + mT1 + cRBF + mRBF	87.23% (74.83–94.02%)	83.33% (71.26–90.98%)	0.87 (0.79–0.95)

*MRI* magnetic resonance imaging, *MN* membranous nephropathy, *HC* healthy control, *AUC* area under the curve, *cT1* cortex T1 mapping, *mT1* medullary T1 mapping, *cRBF* cortex renal blood flow, *mRBF* medullary renal blood flow

### Interobserver variability of MRI parameters measurement

The intraclass correlation coefficient of right kidney length, left kidney length, right cT1, left cT1, right mT1, left mT1, right cRBF, left cRBF, right mRBF, and left mRBF were 0.97 (95% CI, 0.96–0.98), 0.98 (95% CI, 0.98–0.99), 0.82 (95% CI, 0.75–0.88), 0.84 (95% CI, 0.76–0.89), 0.82 (95% CI, 0.74–0.87), 0.82 (95% CI, 0.75–0.88), 0.89 (95% CI, 0.84–0.93), 0.86 (95% CI, 0.80–0.90), 0.86 (95% CI, 0.80–0.91), 0.86 (95% CI, 0.79–0.90), respectively.

## Discussion

To our knowledge, this is the first group-level study using multiparametric MRI to qualitatively assess renal function and blood perfusion status in MN patients. The main findings of this study revealed the value of multiparametric MRI in MN, demonstrating that T1 mapping and ASL could noninvasively detect renal function, perfusion change, and risk stratification in MN. Furthermore, the combination model of T1 and RBF was more capable than single-parameter models for distinguishing the MN from the HC. The results revealed that multiparametric MRI was a potentially useful imaging tool for identifying MN.

T1 mapping, which reflects the molecular environment of tissues and water content, enables assessment of tissue fibrosis [22, 23]. The increased cT1 value indicated the presence of extracellular fluid, which could be a consequence of inflammation, cellular swelling, or interstitial edema [11] or fibrosis resulting from the association of collagen with supersaturated hydrogel [24]. Previous studies have demonstrated that the cortical T1 values in CKD patients were significantly higher than healthy volunteers [12, 22, 25]. Consistent with these findings, our study revealed that MN patients had higher cT1 and mT1 values in the bilateral kidney compared to the HC group. And cT1 values increased gradually with the aggravation of MN. The result suggested that cT1 may serve as a noninvasive biomarker for assessing fibrosis in MN. However, there was no significant difference in mT1 value between mild and moderate to severe groups, which may be related to the relatively complex structure and function of the renal medulla. This phenomenon aligns with findings by Mao et al [26], which suggested that the specificity of mT1 for diagnosing MN may be lower than that of cT1.

ASL has been proven to be an accurate technique for evaluating tissue microvascular perfusion [27, 28]. Recent studies have shown that ASL effectively detects differences in renal perfusion between HC and CKD patients at various disease stages [11, 14, 16, 29]. In our study, RBF data analysis showed that cRBF and mRBF values in patients with the moderate to severe group were significantly lower than the HC group. But there was no significant difference in cRBF and mRBF values between the HC group and the mild group. The result was understandable. In the early stage, MN mainly deposited immune complexes outside the glomerular basement membrane, and the glomerular capillary loops were normal or had little influence on blood perfusion. With the progression of the disease, the diffuse thickening of the basement membrane compresses the capillary lumen, and renal interstitial fibrosis might destroy the microvascular network, eventually leading to decreased renal perfusion [30, 31]. It suggested that RBF could serve as a noninvasive indicator of MN progression.

The pathological characteristics of fibrosis progression in glomerular disease include peritubular capillary remodeling and decreasing [32, 33]. Quantification of peritubular capillary density can reflect the severity of disease and blood perfusion status [34]. In this study, the cT1 average value and RBF value had a significant correlation with eGFR. The results further supported that cT1 and RBF could reflect the renal functional changes of MN, similar to the results in the CKD study [11]. Additionally, the diagnostic efficacy of multiparametric MRI models was significantly higher than the other three single-parameter models. The results indicated that multiparametric MRI models had potential as a noninvasive method to diagnose MN.

Renal biopsy, as the gold standard for diagnosing MN, is not suitable for serial evaluation. Our study revealed the potential of multiparametric MR noninvasive assessment of MN, offering an alternative for the clinical management of MN and compensating for the limitations of invasive methods.

However, there are several limitations in this study. First, the single-center design and small sample size, especially in the moderate to severe group, may affect the clinical applicability of the conclusion. Second, some confounding variables from the HC group were not collected, and therefore, only age and gender were adjusted. Third, this was a cross-sectional study, and it did not reflect a causal relationship between MRI parameters and the progression of MN patients. Finally, the current research cannot precisely distinguish MN, IgA, and diabetic nephropathy. Future research will include more pathological types of CKD. It is necessary to carry out multicenter studies with large cohorts to explore the relevant mechanisms combined with molecular biology techniques and explore the value of multiparametric MRI in monitoring therapeutic efficacy through longitudinal studies.

In conclusion, our findings indicated that a multiparametric MRI approach combining T1 mapping and RBF was a potent noninvasive tool for assessing renal dysfunction and blood perfusion in MN patients. This practical, noninvasive method offers a possibility for assessing risk stratification and dynamically monitoring therapeutic efficacy, thereby improving the clinical management for patients.

## Data Availability

The datasets generated and/or analyzed during the current study are available from the corresponding author on reasonable request.

## Abbreviations

ASL: Arterial spin labeling
AUC: Area under the curve
CKD: Chronic kidney disease
cRBF: Cortical RBF
cT1: Cortical T1 mapping
eGFR: Estimated glomerular filtration rate
FOV: Field-of-view
HC: Healthy controls
ICCs: Intraclass correlation coefficients
MN: Membranous nephropathy
mRBF: Medullary RBF
MRI: Magnetic resonance imaging
mT1: Medullary T1 mapping
RBF: Renal blood flow
ROI: Region of interest
Scr: Serum creatinine
TE: Echo time
TR: Repetition time
